# Fullerene Negative Ions: Formation and Catalysis

**DOI:** 10.3390/ijms21093159

**Published:** 2020-04-30

**Authors:** Zineb Felfli, Kelvin Suggs, Nantambu Nicholas, Alfred Z. Msezane

**Affiliations:** Department of Physics and CTSPS, Clark Atlanta University, Atlanta, GA 30314, USA; zfelfli@cau.edu (Z.F.); kelvlorenz@yahoo.com (K.S.); nantambu.nicholas@students.cau.edu (N.N.)

**Keywords:** fullerene anions, electron cross sections, polarization interaction, water oxidation, anionic catalysis

## Abstract

We first explore negative-ion formation in fullerenes C_44_ to C_136_ through low-energy electron elastic scattering total cross sections calculations using our Regge-pole methodology. Then, the formed negative ions C_44_**ˉ** to C_136_**ˉ** are used to investigate the catalysis of water oxidation to peroxide and water synthesis from H_2_ and O_2_. The exploited fundamental mechanism underlying negative-ion catalysis involves hydrogen bond strength-weakening/breaking in the transition state. Density Functional Theory transition state calculations found C_60_**ˉ** optimal for both water and peroxide synthesis, C_100_**ˉ** increases the energy barrier the most, and C_136_**ˉ** the most effective catalyst in both water synthesis and oxidation to H_2_O_2_.

## 1. Introduction 

To celebrate the International Year of the Periodic Table, the Royal Society of Chemistry published the themed collection ‘Single Atoms as Active Catalysts’ [1]. This has motivated the present investigation of using single fullerene molecular anions as catalysts. Toward this end, we first investigate the formation of negative ions in the fullerene molecules C_44_, C_60_, C_70_, C_98_, C_112_, C_120_, C_132_, and C_136_ through low-energy electron elastic scattering total cross sections (TCSs) calculations. Our robust Regge-pole methodology is used for the calculations. The formed anionic fullerenes C_44_**ˉ** to C_136_**ˉ** during the collisions are then used to investigate the catalysis of water oxidation to peroxide and water synthesis from H_2_ and O_2_. Negative ion catalysis involves anionic molecular complex formation in the transition state, with the atomic negative ion weakening/breaking the hydrogen bond strength. This is the same fundamental mechanism that underlies the well-investigated muon-catalyzed nuclear fusion using a negative muon, a deuteron, and a triton; it has been proposed to drive nanoscale catalysis [2,3]. Specifically, in the experiments [4,5,6], the fundamental atomic mechanism responsible for the oxidation of water to peroxide catalyzed by Au and Pd nanoparticles has been attributed to the interplay between Regge resonances and Ramsauer–Townsend (R-T) minima in the electron elastic TCSs for the Au and Pd atoms, along with their large electron affinities (EAs) [2,3]. 

The mechanism of negative-ion catalysis has been demonstrated in the oxidation of H_2_O to H_2_O_2_ catalyzed using the Au**ˉ** and Pd**ˉ** anions to understand the experiments of Hutchings and collaborators [4,5,6], in the catalysis of light, intermediate and heavy water to the corresponding peroxides [7], and in the oxidation of methane to methanol without the CO_2_ emission [8] to name a few. Briefly, the experiments [4,5,6] synthesized hydrogen peroxide from H_2_ and O_2_ using supported on Fe_2_O_3_ Au, Pd, and Au-Pd nanoparticles as catalysts. Importantly, these experiments found that the addition of Pd to the Au catalyst increased the rate of H_2_O_2_ synthesis significantly as well as the concentration of the formed H_2_O_2_. In [4], it was found that the production of H_2_O_2_ increased 7- and 30-fold over that of the Au catalyst alone when using the Pd and Au-Pd, respectively. Recently, the experiment [6] used the less expensive atomic Sn catalyst for possible water purification in the developing world. Consequently, here we explore the effectiveness of the fullerene negative ions C_44_**ˉ** to C_136_**ˉ** in the catalysis of water oxidation to peroxide and water synthesis from H_2_ and O_2_ in search of less expensive catalysts. The focus is particularly on the larger fullerene molecules greater than C_70_.

The importance of fullerene molecules in negative ion catalysis, organic solar-cells, sensor technology, drug delivery, catalytic efficiency in fundamental hydrogenation, etc., has motivated us to study the variation of the EA with the fullerene size from C_44_ to C_136_ and contrast the EAs with that of the standard C_60_. Manifesting the existence of long-lived negative ion formation, reliable atomic, and molecular affinities are crucial for understanding the vast number of chemical reactions involving negative ions [9]. In the formation of fullerene negative ions, it has been demonstrated for the first time that the ground state anionic binding energies (BEs) extracted from our Regge-pole calculated TCSs for the C_20_ through C_92_ fullerenes matched excellently the measured EAs [10,11,12,13,14,15,16,17]. This provides a novel and general approach to the determination of reliable EAs for complex heavy systems. Indeed, the EAs provide a stringent test of theoretical calculations when their results are compared with those from reliable measurements. In addition, the Regge-pole methodology requires no assistance whatsoever from either experiment or other theory for the remarkable feat. The results [18,19] provided great credence to the power and ability of the Regge-pole methodology to extract reliable EAs of the fullerene molecules from the calculated ground states electron elastic TCSs. It is noted here that obtaining unambiguous and reliable fullerene EAs is a challenging task for existing theoretical methods. Generally, the Regge-pole calculated low-energy electron elastic TCSs for fullerene molecules are characterized by ground, long-lived polarization-induced metastable, and excited negative ion formation. 

Except for the C_60_ fullerene, theoretical and/or experimental low-energy electron elastic scattering TCSs for fullerenes are generally sparse. For C_60_, low-energy electron scattering cross sections have been investigated theoretically [20,21,22,23,24,25,26,27]. Very recently, angle-differential electron elastic scattering from C_60_ has been studied [28]. The investigations of Wigner Time Delay in electron-C_60_ elastic scattering [29] using potential models defined by the fullerene EA and its radius will certainly benefit from this study. Experimentally, low-energy electron elastic scattering differential cross sections for C_60_ were measured [30]. Gas phase fullerenes C_76_ and C_78_ [31] and gas phase C_60_ and C_70_ [32] have been studied using low-energy electron scattering. In the latter study, several resonant states were identified including the determination of the lifetimes of the formed negative ions. Thermal rate coefficients and cross sections for electron attachment to C_60_ have been studied [33] including their low energy temperature dependence in a crossed electron beam–molecular beam experiment [34].

The low-energy electron elastic collision TCSs of the fullerene molecules obtained in this paper as well as those of the already studied fullerenes [18,19,35,36] and the actinide [37] and the lanthanide [38,39] atoms should contribute to a better understanding of the role of the individual atoms/fullerenes in ongoing studies involving endohedral systems [40,41,42,43,44,45,46]. Additionally, expected to benefit from this study will be the exploration of the M@C_60_ (M = Ti, Zr,U) fullerene hybrids that have demonstrated catalytic efficiency in fundamental hydrogenation [47]. 

## 2. Results 

In Section 2.1 we first present the variation with the electron impact energy E of the Regge-pole calculated electron elastic scattering TCSs for the fullerene molecules C_44_ to C_136_. Section 2.2 demonstrates the utility of the fullerene molecular anions in the catalysis of water oxidation to peroxide and water synthesis from H_2_ and O_2_ using the anionic fullerene catalysts C_44_**ˉ** to C_136_**ˉ**.

### 2.1. Fullerene Electron Scattering Total Cross Sections

In fullerene negative ion formation, it has been demonstrated for the first time that the ground state anionic BEs extracted from our Regge-pole calculated electron elastic scattering TCSs for the C_20_ through C_92_ fullerenes matched excellently the measured EAs of these fullerenes [18,19]. This provides a novel and general approach to the determination of unambiguous and reliable EAs for complex heavy systems. The Regge-pole methodology requires no assistance whatsoever from either experiment or other theory to achieve the remarkable feat. 

**Figure 1:**
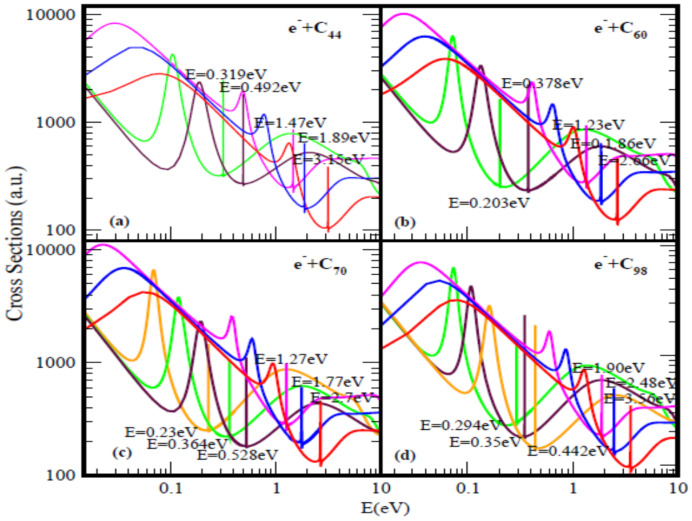
Total cross sections (a.u.) for (**a**) C_44_, (**b**) C_60_, (**c**) C_70_ and (**d**) C_98_. The red, blue and pink curves represent total cross sections (TCSs) for the ground and induced metastable states (first and second), respectively. The green and brown curves in (**a**) and (**b**) denote the TCSs for the first and the second excited states, respectively. For C_70_ and C_98_ the orange, green and brown curves represent the excited states TCSs. The dramatically sharp resonances correspond to the fullerene anions formed during the collisions.

**Figure 2:**
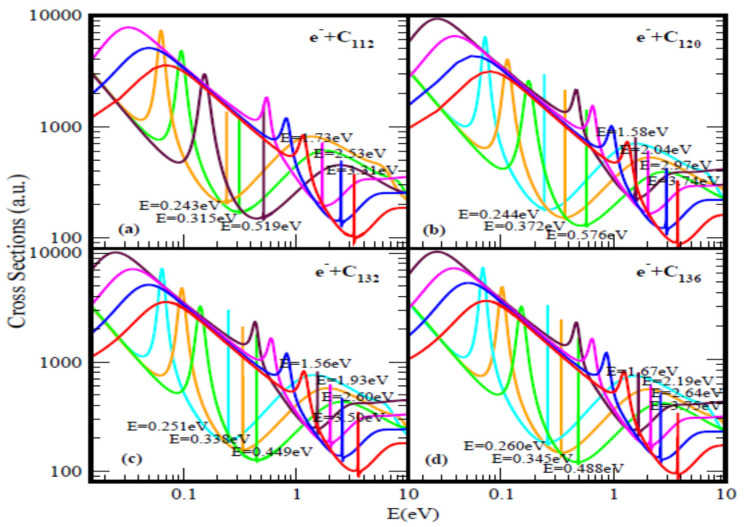
Total cross sections (a.u.) for (**a**) C_112_, (**b**) C_120_, (**c**) C_132_ and (**d**) C_136_. The red, blue, pink and brown (no brown curve for C_112_) curves represent TCSs for the ground and induced metastable states (first, second and third), respectively. For C_112_ (orange, green and brown), while for C_120_, C_132_ and C_136_ (light blue, orange and green) curves correspond to the excited TCSs. The dramatically sharp resonances correspond to the anions formed during the collisions.

Figure 1 and Figure 2 present the elastic TCSs for the fullerene molecules C_44_ through C_136_ and Table 1 summarizes the essential data. Indeed, the Regge-pole calculated low-energy electron elastic TCSs for the fullerene molecules considered here are found to be characterized generally by ground, polarization-induced metastable and excited negative ion formation. For ground state collisions the resultant anionic BEs yield the theoretically challenging to calculate EAs and demonstrate their wide variation from fullerene to fullerene. The results here are consistent with the observation that low-energy electron-fullerene interactions are generally characterized by rich resonance structures [32,48,49,50] and that the experimentally detected fullerene isomers correspond to the metastable states [51]. They also support the conclusion that the EAs of fullerene molecules are relatively large [52]. This should satisfy part of the requirement to increase fullerene acceptor resistance to degradation by the photo-oxidation mechanism as well as improve the understanding of the degradation mechanism in organic solar cells [53]. The determined EAs here could also be employed to construct the widely used simple model potentials for the fullerene shells, including endohedral fullerenes [54]. The resonance-rich structures of the fullerene TCSs and their large EAs explain the tendency of fullerenes to form compounds with electron-donor anions and their vast applications as well. These TCSs require careful delineation and identification of the attendant resonance structures for reliable interpretation as well as extraction of the EAs.

For a better appreciation of the physics underlying the resonance-rich TCSs for the various fullerenes presented in the Figure 1 and Figure 2, we first discuss briefly the TCSs for the C_44_ fullerene. With less structure, the TCSs were first calculated in [18]; here they have been recalculated to expose more resonances. It is noted that generally the internal region of zero potential provided by the hollow cage structure of the fullerenes is conducive to metastable anionic formation during the collisions. This is clearly manifested through the appearance of additional resonances in the TCSs as the fullerene size increases from C_44_ through C_136_. Also, this explains the existence of the two series of resonances, the first is associated with the ground state TCS while the second belongs to the highest excited state TCS (green curve). 

Focusing specifically on the C_44_ TCSs, Figure 1a, the red, blue, pink, brown and green curves represent respectively the TCSs of the ground; the first & the second metastable and the two excited states. The fundamental physics underlying these curves can be readily understood if we focus on each color-coded TCS. For the analysis we select the ground state TCS curve, the red curve. Near threshold the TCS exhibits the characteristic shape resonance (SR), broad maximum. As the electron energy is increased, the fullerene becomes polarized and reaches maximum polarization manifested through the appearance of the first R-T minimum at about 1.01 eV, indicative that the polarization interaction has been accounted for adequately in the calculation [60]. With further increase in the electron impact energy, the electron becomes trapped by the centrifugal potential, demonstrated by the appearance of the SR at 1.41 eV. As the electron leaks out of the centrifugal potential, the C_44_ shell, due to its strong polarizability, becomes significantly polarized leading to the generation of the second deep R-T minimum in the TCS at 3.13 eV. At the absolute minimum the long-lived ground state of the C_44_ˉ anion is formed with the BE of 3.15 eV. At the R-T minimum the electron spends many angular rotations about the C_44_ as it decays; the angular life is determined by 1/[Im λ*_n_*(E)] → ∞, since for the ground state resonance Im λ*_n_*(E) → 0, see Equation (1). Notably, at the R-T minimum new molecules can be created from fermions.

The analysis is also applicable to the other fullerene TCSs presented in Figure 1 as well as in Figure 2. The extracted BEs of the negative ions formed during the collisions are summarized in Table 1 where they are compared with available EAs. Indeed, for the ground state collisions the extracted from the TCSs anionic BEs correspond to the EAs of the fullerenes. The Regge-pole calculated TCSs for the C_60_ fullerene presented in Figure 1b is taken from [39]. The TCSs, typical of those calculated in this paper, are found to be characterized generally by dramatically sharp resonances manifesting ground, metastable and excited anionic formation during the collisions, Ramsauer-Townsend (R-T) minima and shape resonances. Indeed, the ground state TCS (red curve) yields the anionic BE, located at its absolute R-T minimum; it has been identified with the C_60_ EA [19]. Viewed as presented in the Figure 1b the C_60_ TCSs appear complicated as well. However, they are readily understood and interpreted as was done in [19,39]. This ground state TCS is clearly shown alone in Figure 1 of [19] and the underlying physics is also presented there. 

Figure 1b and Figure 2 demonstrate the variation of the electron TCSs with E for the C_60_, C_70_, C_98_, C_112_, C_120_, C_132_ and C_136_ fullerene molecules. Clearly, these TCSs are characterized as in the C_44_ case by ground, metastable and excited anionic formation, R-T minima and shape resonances. The extracted anionic BEs from the ground states TCSs correspond to the EAs of the fullerene molecules. These BEs, presented in Table 1 demonstrate their wide variation from fullerene to fullerene. The various dramatically sharp resonances in the TCSs represent negative ion formation in the ground, metastable and excited states. 

### 2.2. Fullerene Transition State Barriers 

The utility of the fullerene negative ions has been demonstrated in the catalysis of water oxidation to peroxide and water synthesis from H_2_ and O_2_ using the anionic fullerene catalysts C_44_**ˉ** to C_136_**ˉ**. The reactions of interest are:

Water Oxidation to Peroxide Reaction:

2H_2_O + O_2_ → 2H_2_O_2_; (1)

Water Synthesis Reaction:

2H_2_ + O_2_ → 2H_2_O. (2)

Reaction (1) is similar to Equation (1) of Ref. [2] where the active catalyst is the Auˉ anion. The processes considered here are exactly similar to that, except that here the Auˉ anion catalyst is replaced by the C_44_**ˉ** to C_136_**ˉ** anion catalysts. We will therefore use the familiar Auˉ anion catalyst to explain and demonstrate the importance of the transition state (TS) in the reactions. Additionally, in the end, we will simply replace the Auˉ with the C_44_**ˉ** to C_136_**ˉ** anion catalysts. Since the final product, viz. Equation (4) of Ref [2] is devoid of the catalyst as it should, we look at the transition states, Equation (2), and Equation (1). In the oxidation of H_2_O to H_2_O_2_ catalyzed by the Auˉ anion, the anion–molecular complex Au**ˉ**(H_2_O)_1,2_ is formed in the TS. This complex subsequently breaks up into Au**ˉ** and (H_2_O)_1_ and (H_2_O)_2_. The large EA of atomic Au played an essential role in the process. It is important in the dissociation energy of the complex Au**ˉ**(H_2_O)_1,2_ into the above products. The need in negative ion catalysis for systems with reliable EAs is now evident. In the present calculation, we simply replace the Au**ˉ** anion catalyst with the fullerene anion catalysts.

**Figure 3:**
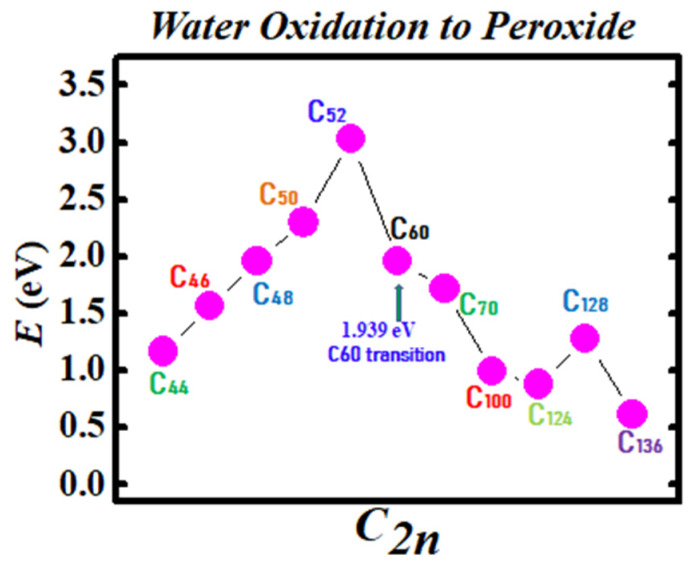
Transition state calculation of anionic fullerenes C_44_**ˉ** to C_136_**ˉ** catalyzing water oxidation to peroxide.

**Figure 4:**
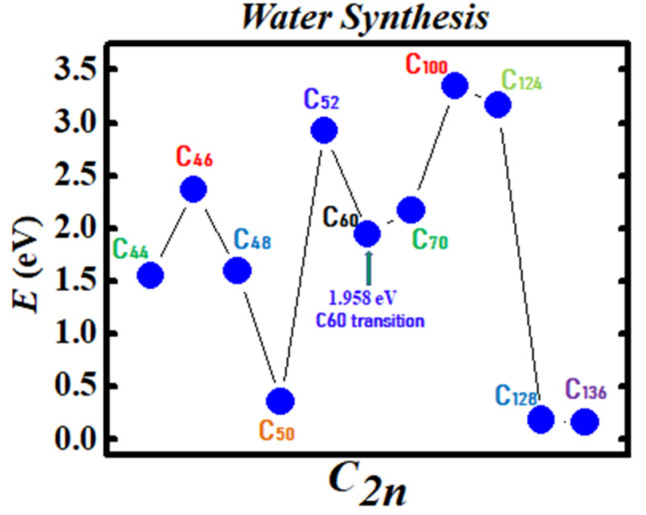
Transition state calculation of anionic fullerenes C_44_**ˉ** to C_136_**ˉ** catalyzing oxygen and hydrogen synthesis to water.

Figure 3 and Figure 4 demonstrate the Density Functional Theory (DFT) calculated transition states. DFT and dispersion corrected DFT approaches have been employed for the transition state evaluations. Geometry optimization of the structural molecular conformation utilized the gradient-corrected Perdew-Burke-Ernzerhof parameterizations [61] of exchange-correlation as implemented in DMol3 [62]. A tolerance of 1x10^-3^ Ha was used with a smearing value of 0.005 Ha. DFT calculated energy barriers reduction in the oxidation of H_2_O to H_2_O_2_ catalyzed using the anionic fullerene catalysts C_44_**ˉ** to C_136_**ˉ** are shown in Figure 3. Results in Figure 4 are for the water synthesis from H_2_ and O_2_ catalyzed using the anionic fullerene catalysts C_44_**ˉ** to C_136_**ˉ** as well. 

DFT transition state calculations found the C_52_**ˉ** and C_60_**ˉ** anions to be numerically stable for both water oxidation and water synthesis and the C_100_**ˉ** anion to increase the energy barrier the most in the water synthesis from H_2_ and O_2_. When catalyzing both water oxidation to peroxide and synthesis from H_2_ and O_2_, the C_136_**ˉ** anion has proved to be the most effective in reducing the energy barrier significantly. Importantly, a single large fullerene such as the C_136_, C_120_, or even the C_70_ could replace the Au, Pd, and Sn atoms in the catalysis of H_2_O_2_ from H_2_O in the experiments of Hutchings and collaborators [4,5,6] acting as a multiple-functionalized catalyst. These fullerenes have their metastable BEs close to the EAs of the used atoms in the experiments. Thus, an inexpensive dynamic water purification system for the developing world could be realized [6]. 

## 3. Method of Calculation

In [63] it was confirmed that Regge poles formed during low-energy electron elastic scattering become stable bound states. Here we adopt the Regge-pole methodology, also known as the complex angular momentum (CAM) method for the calculation of the electron scattering TCSs. Regge poles, singularities of the S-matrix, rigorously define resonances [64,65]. Being generalized bound states, they can be used to calculate reliably the anionic BEs of the ground, metastable and excited states of complex heavy systems through the TCSs calculations. The Mulholland formula [66] is used here to calculate the near-threshold electron–fullerene collision TCS resulting in negative ion formation as resonances. In the form below, the TCS fully embeds the essential electron-electron correlation effects [67,68] (atomic units are used throughout):

(3) $$\sigma_{tot}\left(E\right)=4\pi k^{-2}\int_{0}^{\infty }Re\left[1-S\left(\lambda \right)\right]\lambda d\lambda -8\pi^{2}k^{-2}\sum_{n}Im\frac{\lambda_{n}\rho n}{1+\exp(-2\pi i\lambda_{n})}+I\left(E\right)$$

In Equation (3) S(λ) and λ are respectively the S-matrix and the CAM, $k=\;\sqrt{2mE}$, *m* being the mass and *E* the impact energy, *ρ_n_* is the residue of the S-matrix at the *n*^th^ pole, λ*_n_* and *I*(*E*) contains the contributions from the integrals along the imaginary λ-axis; its contribution has been demonstrated to be negligible [69].

As in [26] the complicated details of the electronic structure of the fullerene itself are not considered here. The incident electron is assumed to interact with the complex atom/fullerene through the Thomas-Fermi type potential, known as the Avdonina, Belov and Felfli (ABF) potential [70] which accounts for the vital core-polarization interaction

(4) $$U\left(r\right)=\frac{Z}{r\left(1+\alpha z^{\frac{1}{3}}r\right)(1+\beta z^{\frac{2}{3}}r^{2})}$$

In Equation (4) *Z* is the nuclear charge, *α* and *β* are variation parameters. This potential has the appropriate asymptotic behavior, *viz.* ~ −1/(αβr^4^) and accounts properly for the polarization interaction at low energies. This potential, extensively studied [71], has five turning points and four poles connected by four cuts in the complex plane. The presence of the powers of Z as coefficients of *r* and *r*^2^ in Equation (4) ensures that spherical and non-spherical atoms/fullerenes are correctly treated. The effective potential $V\left(r\right)=U\left(r\right)+\lambda \left(\lambda +1\right)/2r^{2}$ is considered here as a continuous function of the variables *r* and complex *λ*. The details of the numerical evaluations of the TCSs have been described in [68] and further details of the calculations may be found in [72]. 

In the calculations, the optimal value of *α* was determined to be 0.2. When the TCS as a function of *β* has a dramatically sharp resonance [69], corresponding to the formation of a stable negative ion, this resonance is longest lived for a given value of the energy, which corresponds to the EA of the system (for ground state collisions) or the BE of the metastable/excited anion. Also calculated in the CAM methods are the Regge Trajectories, viz. *Im* λ*_n_*(E) versus *Re* λ*_n_*(E); they have been used to demonstrate that at low energy relativistic and non-relativistic calculations yield the same results [73]. 

## 4. Conclusions

The Regge-pole calculated low-energy electron elastic TCSs for the fullerene molecules considered here are found to be characterized generally by ground, metastable, and excited negative ion formation. Indeed, the rich resonance structures of the fullerenes TCSs and their large EAs explain the tendency of fullerenes to form compounds with electron-donor anions and their vast applications as well.

The utility of the formed negative ions has been demonstrated in the catalysis of water oxidation to peroxide and water synthesis from H_2_ and O_2_ using the anionic fullerene catalysts C_44_**ˉ** to C_136_**ˉ**. Transition state calculations using DFT found the C_52_**ˉ** and C_60_**ˉ** anions to be robust (yielding essentially the same transition state energies) for both water and peroxide synthesis and the C_136_**ˉ** to be the most effective in reducing the energy barrier significantly. Importantly, a single large fullerene such as the C_136_, C_120_, or even the C_60_ could replace the Au, Pd, and Sn atoms in the catalysis of H_2_O_2_ from H_2_O in the experiments [4,5,6] acting as a multiple-functionalized catalyst. Thus, an inexpensive dynamic water purification system could be realized through the use of fullerene anions as catalysts. Furthermore, these fullerenes could also be used as catalysts in the production of methanol from methane without carbon dioxide emission with significant impact on the environment.

## Figures and Tables

**Table 1:** Fullerene ground (GR-S), metastable (MS-n, n = 1, 2, 3) and first excited (EXT-1), second excited (EXT-2) and third excited (EXT-3) anionic states binding energies (BEs). R-T refers to the energy position of the ground state R-T minimum. The measured EAs are represented as Expt. All the energies are in eV.

Full.	Bes GR-S	BEs MS-1	BEs MS-2	BEs MS-3	BEs EXT-1	BEs EXT-2	BEs EXT-3	R-T GR-S	EA Expt.
C_44_	3.15	1.89	1.47	-	0.319	0.492	-	3.13	3.30 [15]
C_60_	2.663 [19] 2.57 [55] 2.23 [56] 2.63 [57]	1.86	1.23	-	0.203	0.378	-	2.68	2.65 [10] 2.666 [12] 2.664 [58] 2.684 [11]
C_70_	2.70	1.77	1.27	-	0.230	0.364	0.528	2.72	2.676 [12] 2.72 [14] 2.765 [13] 2.74 [59]
C_98_	3.56	2.48	1.90	-	0.294	0.350	0.442	3.54	-
C_112_	3.31	2.53	1.73	-	0.243	0.315	0.519	3.32	-
C_120_	3.74	2.97	2.04	1.58	0.244	0.372	0.576	3.73	-
C_132_	3.59	2.60	1.93	1.56	0.251	0.338	0.449	3.58	-
C_136_	3.75	2.64	2.19	1.67	0.260	0.345	0.488	3.77	-
